# The association between nicotine stomatitis and waterpipe smoking

**DOI:** 10.18332/tid/189600

**Published:** 2024-06-27

**Authors:** Hussain Dashti, Devipriya Sundaram

**Affiliations:** 1 Department of Diagnostic Sciences, College of Dentistry, Kuwait University, Safat, Kuwait

**Keywords:** nicotine stomatitis, waterpipe, smoking, hot beverages, shisha

## Abstract

**INTRODUCTION:**

Waterpipe smoking (WPS) is gaining popularity worldwide, especially in the Middle East, and significantly affects oral health. Nicotine stomatitis (NS) is an inflammatory response of the palate to the heat and chemical irritation caused by tobacco. The high temperatures of hot beverages have been found to have a synergistic effect. This study investigated the association of NS among waterpipe smokers and hot beverage drinkers.

**METHODS:**

This cross-sectional study was conducted in several public locations in Kuwait. Demographic data, smoking habits, and hot beverage intake were recorded using questionnaires. An oral examination was performed with informed consent, and the occurrence of NS was recorded. Data were analyzed using IBM SPSS statistics version 28.0 (IBM Inc., Chicago, IL, USA).

**RESULTS:**

Of the 211 participants, 55 subjects (26.1%) presented with NS. All the patients with NS drank hot beverages, while 37 (67.3%) patients with NS were waterpipe smokers and hot beverage drinkers. Smokers with NS smoked significantly more tobacco heads (Z= -2.606; p=0.009) and for more hours per day (Z= -2.222; p=0.026).

**CONCLUSIONS:**

This study explored the association between WPS and NS in Kuwait. Waterpipe smokers and males were more likely to present with NS. Also, the number of tobacco heads and the number of hours of WPS were found to correlate with the presence of lesions. Effective strategies to reduce WPS need to be implemented. Further studies are recommended to investigate the cause-andeffect relationships.

## INTRODUCTION

Waterpipe smoking (WPS) is an ancient form of smoking tobacco that originated in Persia and spread to the Middle East^1^. WPS is recognized worldwide by many names depending on the region, country, and culture^2^.

A systematic review of medical literature on waterpipe tobacco prevalence and trends concluded that the prevalence among adults was highest in the Eastern Mediterranean region, while the highest prevalence among youth was found in both Eastern Mediterranean and European regions^3^. Data from around the world shows that even Western societies have not been spared by WPS, which has become a global epidemic^4^.

Accurate prevalence estimates of Shisha-only smoking are challenging because the target populations in various studies differ. Smoking is widely prevalent in Kuwait, and shisha smoking is as popular as cigarette smoking^5^. Shisha is one of the most common modalities used to indulge in tobacco products, especially among females, in Kuwait^6^. Furthermore, a self-reported questionnaire study about smoking behavior and beliefs among a non-random sample of Kuwaitis found that 45% smoked only waterpipe^7^.

Nicotine is a main chemical present in all forms of tobacco and is found in varying amounts (<0.01–9.29 mg of nicotine per session) depending on the type of tobacco used in waterpipe smoking^8^. WPS has been attributed to paving the way to nicotine addiction. Daily waterpipe usage has been found to produce nicotine absorption of a magnitude similar to that of daily cigarette usage at a rate of 10 cigarettes per day^9^.

Several studies have investigated the effects of waterpipes on oral health, demonstrating that this type of tobacco may have negative consequences on the oral mucosa and the periodontium^10^. Nicotine stomatitis (NS) is an oral lesion usually located in the hard palate, which develops due to heat and chemical irritation from tobacco products, commonly generated by tobacco smoking. Drinking hot beverages has been found to have a synergistic effect with tobacco smoking^11^. In addition, NS has been reported in non-smoking individuals who drink extremely hot beverages, suggesting that heat alone can induce NS^11,12^. NS presents as a white/gray-thickened oral mucosa with numerous slightly raised papules centered by punctate red dots. The thickened white/ grey mucosa is due to hyperkeratosis, while elevated papules with punctate red centers represent inflamed minor salivary glands with ductal orifices. Moreover, hyperkeratosis and inflamed minor salivary glands are oral responses that correlate in severity with the duration and level of heat and nicotine exposure^13,14^. These mucosal changes are most often observed in pipe and reverse cigarette smokers^15^. The condition has also been noted in electronic cigarette users^16^.

In Kuwait, the frequency of NS was found to be higher among smokers than among non-smokers^17^. However, to the best of our knowledge, the oral findings of NS in waterpipe smokers have not been studied. Therefore, this study aimed to investigate the association of NS in waterpipe smokers and hot beverage drinkers.

## METHODS

A cross-sectional study was conducted from September 2019 to February 2020 among a convenient sample of adults aged >21 years. The sample size was estimated using G*Power software (latest version 3.1.9.7; Heinrich-Heine-Universität Düsseldorf, Düsseldorf, Germany). Using a conservative effect size of 0.2, for an error of 5% and a power of 80%, a minimum of 199 subjects would be required. Participants were recruited from the local community in Kuwait, including dental clinics, cafés, social gatherings, and other public places. Subjects who had been regular waterpipe smokers for at least one year were categorized as smokers. Participants who had never used tobacco in any form or had smoked conventional cigarettes earlier than the last six months were considered non-smokers. As NS is a reversible condition, six months is a convenient period to reverse any changes caused by conventional cigarettes. Participants who smoked other forms of tobacco, such as conventional cigarettes, cigars, and e-cigarettes, along with waterpipes, were excluded from the study.

This study was approved by the HSC Ethics Committee of Kuwait University with the Reference number VDR/EC/3450. Patient consent was obtained prior to the commencement of the study.

Basic data on the participants, such as age, sex, nationality, and education level, were collected. Questions regarding the intake of hot beverages (type, frequency, amount, and perceived heat rate) were also included. Data on the frequency of smoking, perceived heat rate, preferred brand, flavor, and the number of tobacco heads consumed were also collected for shisha smokers. All the above information was collected using a self-administered questionnaire written in both English and Arabic.

**Figure 1:**
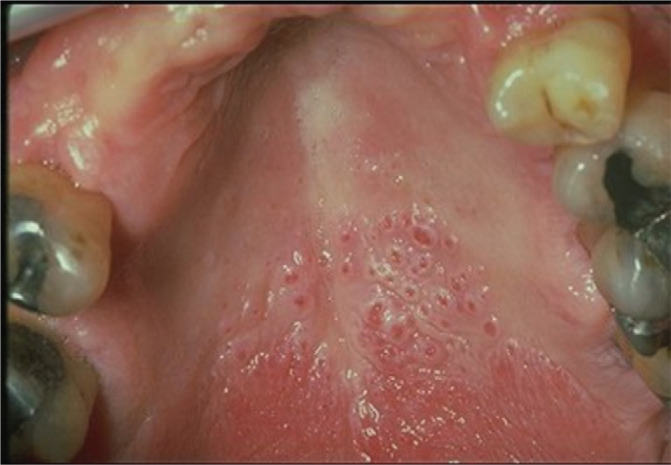
Patient with nicotine stomatitis

After obtaining the patient’s consent, the main investigator performed an oral examination of all participants’ palates, where only a dental mirror and torch were used to inspect the condition of the palate. Photographs of the participants’ oral cavities were obtained as required (Figure 1).

To the best of our knowledge, standardized objective measures of NS have not been documented. Previous studies have developed grading criteria for oral stomatitis and mucositis; however, these criteria are not specific to NS. These publications include the National Cancer Institute’s Common Toxicity Criteria (NCI CTC) and the WHO’s Oral Toxicity Scale (OTS)^18-20^. The grading criteria for NS observed among reverse smokers have also been published^21^. These grading systems focused on premalignant lesions and other clinical symptoms, such as pain and inability to swallow, which would lead to impaired nutritional status and inadequate hydration, in addition to the proper medical intervention required. Therefore, we developed a criterion, as shown in (Supplementary file Table 1) based on previously established grading systems for NS.

Data analyses were conducted using IBM SPSS statistics version 28.0 (IBM Inc., Chicago, IL, USA). Descriptive statistics (frequencies and percentages) were calculated, and associations between categories were assessed using the chi-squared test. Logistic regression analysis was performed to analyze the relationship between the independent variables and the occurrence of NS. The Mann-Whitney U-test was used to compare differences in non-normally distributed ordinal variables between patients with or without NS. Statistical significance was set at p<0.05.

## RESULTS

In total, 211 individuals consented to participate in this study. Table 1 shows the demographic characteristics of participants. Of the 211 participants, 100 (47.4%) were male. Approximately 50.5% (53 out of 105) of waterpipe smokers belonged to the group aged 21–30 years, and the percentage of smokers gradually decreased with an increase in age. There was a significant association between age and smoking status (p<0.001). In addition, 68.6% (72 out of 105) of waterpipe smokers were Kuwaitis, while most non-smokers (61.3%) belonged to the expatriate population. A statistically significant difference (p<0.001) was observed between nationalities and smoking status. Only 7 of the 211 participants did not drink any hot beverages, and 100 (95.2%) drank hot beverages as well as smoked shisha.

**Table 1:** Demographic characteristics of the study participants (N=211)

*Characteristics*	*Non-smokers (N=106) n (%)*	*Smokers (N=105) n (%)*
**Gender**		
Male	50 (47.2)	50 (47.6)
Female	56 (52.8)	55 (52.4)
**Age** (years)*		
21–30	23 (21.7)	53 (50.5)
31–40	36 (34)	34 (32.4)
41–50	30 (28.3)	12 (11.4)
≥51	17 (16)	6 (5.7)
Nationality*		
Kuwaiti	41 (38.7)	72 (68.6)
Expatriate	65 (61.3)	33 (31.4)
**Education level^§^**		
Lower than high school	7 (6.8)	0 (0)
High school	14 (13.6)	21 (20.4)
Higher than high school	82 (79.6)	82 (79.6)
**Hot beverages**		
Drinkers	104 (98.1)	100 (95.2)
Non-drinkers	2 (1.9)	5 (4.8)

§ The total percentage does not equal the exact number, as a few responses were missing.

* p<0.001.

A total of 55 (26.1%) had NS. Table 2 summarizes the findings of the study. Thirty-seven (67.3%) participants with lesions were smokers, whereas 18 (32.7%) were non-smokers. There was a significant difference (p=0.003) in the odds of presenting with NS between shisha smokers and non-smokers (OR=2.66; 95% CI: 1.39–5.07). A similar statistically significant difference (p<0.001) in the odds of lesion prevalence was observed between the sexes. Males were more likely to present with NS (OR=3.03; 95% CI: 1.59–5.79).

**Table 2:** Prevalence of nicotine stomatitis in the study sample with respect to the sociodemographic characteristics

*Characteristics*	*Lesion (N=55) n (%)*	*No Lesion (N=156) n (%)*
Gender**		
Male	37 (67.3)	63 (40.4)
Female	18 (32.7)	93 (59.6)
**Age** (years)		
21–30	21 (38.2)	55 (35.3)
31–40	20 (36.4)	50 (32.1)
41–50	8 (14.5)	34 (21.8)
≥51	6 (10.9)	17 (10.9)
**Nationality**		
Kuwaiti	31 (56.4)	82 (52.6)
Expatriate	24 (43.6)	74 (47.4)
**Education level^§^**		
Lower than high school	2 (3.9)	5 (3.2)
High school	13 (25.5)	22 (14.2)
Higher than high school	36 (70.6)	128 (82.6)
**Hot beverages**		
Drinkers	55 (100)	149 (95.5)
Non-drinkers	0 (0)	7 (4.5)
Smoking status*		
Smokers	37 (67.3)	68 (43.6)
Non-smokers	18 (32.7)	88 (56.4)

§ The total percentage does not equal the exact number, as a few responses were missing.

* p<0.05.

** p<0.001.

All 55 (100%) participants with lesions drank hot beverages, but none of the non-drinkers had lesions. Thirty-seven participants (67.3%) with NS drank hot beverages or smoked waterpipes (Table 3).

**Table 3:** Distribution of patients with/without nicotine stomatitis with respect to hot beverage intake and shisha smoking status

*Smoking status*	*Hot beverage intake*	*Patients with lesions n (%)*	*Patients without lesions n (%)*	*Total n (%)*
**Smokers**	Drinkers	37 (37)	63 (63)	100 (95.2)
	Non-drinkers	0 (0)	5 (100)	5 (4.8)
**Non-smokers**	Drinkers	18 (17.3)	86 (82.7)	104 (98.1)
	Non-drinkers	0 (0)	2 (100)	2 (1.9)
**Total**		55 (26.1)	156 (73.9)	211 (100)

In hot beverage drinkers with NS, the percentage of participants with lesions increased as the number of cups of hot beverage consumed increased. Additionally, a greater number of patients with lesions perceived more heat from their hot beverages (Supplementary file Table 2).

Table 4 shows the smoking characteristics of waterpipe smokers. Mann-Whitney U test showed that there was a significant difference (U=726.500, p=0.009) between the number of tobacco heads per day for the participants with mild NS and participants without the lesion (Table 5). Mann-Whitney U test also showed that there was a significant difference (U=882.000, p=0.026) between the number of hours of smoking shisha per day for the participants with mild NS and participants without the lesion.

**Table 4:** Smoking characteristics of waterpipe smokers

	Waterpipe smokers n (%)*
**Waterpipe smoking** (number of hours per day)	
<1	18 (17.6)
1–2	30 (29.4)
2–3	31 (30.4)
≥4	23 (22.5)
**Perceived heat generated by shisha**	
Mild	13 (12.9)
Moderate	73 (72.3)
High	15 (14.9)
**Number of tobacco heads per day**	
<1	13 (12.9)
1	73 (72.3)
2	15 (14.9)
≥3	21 (21.9)

* The total percentage does not equal the exact number, as a few responses were missing.

**Table 5:** Non-parametric test showing the differences in effects of smoking characteristics in hookah smokers with or without lesions

*Smoking characteristics*	*Presence or absence of nicotine stomatitis*	*n*	*Mean rank*	*Sum of ranks*	*Mann Whitney U*	*Z statistic*	*p*
**Number of hours per day**	Lesion	36	60.00	3093.00	882.000	-2.222	0.026*
	No Lesion	66	46.86	2160.00			
**Number of tobacco heads per day**	Lesion	34	58.13	1976.50	726.500	-2.606	0.009*
	No Lesion	62	43.22	2679.50			

* p<0.05.

The results of our study showed that the mean hours of shisha smoking per day were significantly higher (Z= -2.222; p=0.026) among smokers with lesions than among smokers without lesions at a significance level of 0.05. Smokers with NS smoked a significantly higher number of tobacco heads (Z= -2.606; p=0.009) at a significance level of 0.05.

‘Fakher’ was the most preferred hookah brand by smokers in this study (n=14). Moreover, grape was the favorite flavor (21.4%). In total, 20.4% of the participants preferred both grape and mint. The vast majority (53.1%) of hot beverage drinkers (110 of 204) drank both coffee and tea, while 47 (23%) and 41 (20.1%) drank coffee and tea, respectively. In addition to coffee and tea, other types of hot beverages include a wide variety of herbal teas (ginseng, ginger, lemon, anise, cardamom, and chamomile), hot milk, and hot chocolate.

## DISCUSSION

**Figure 2:**
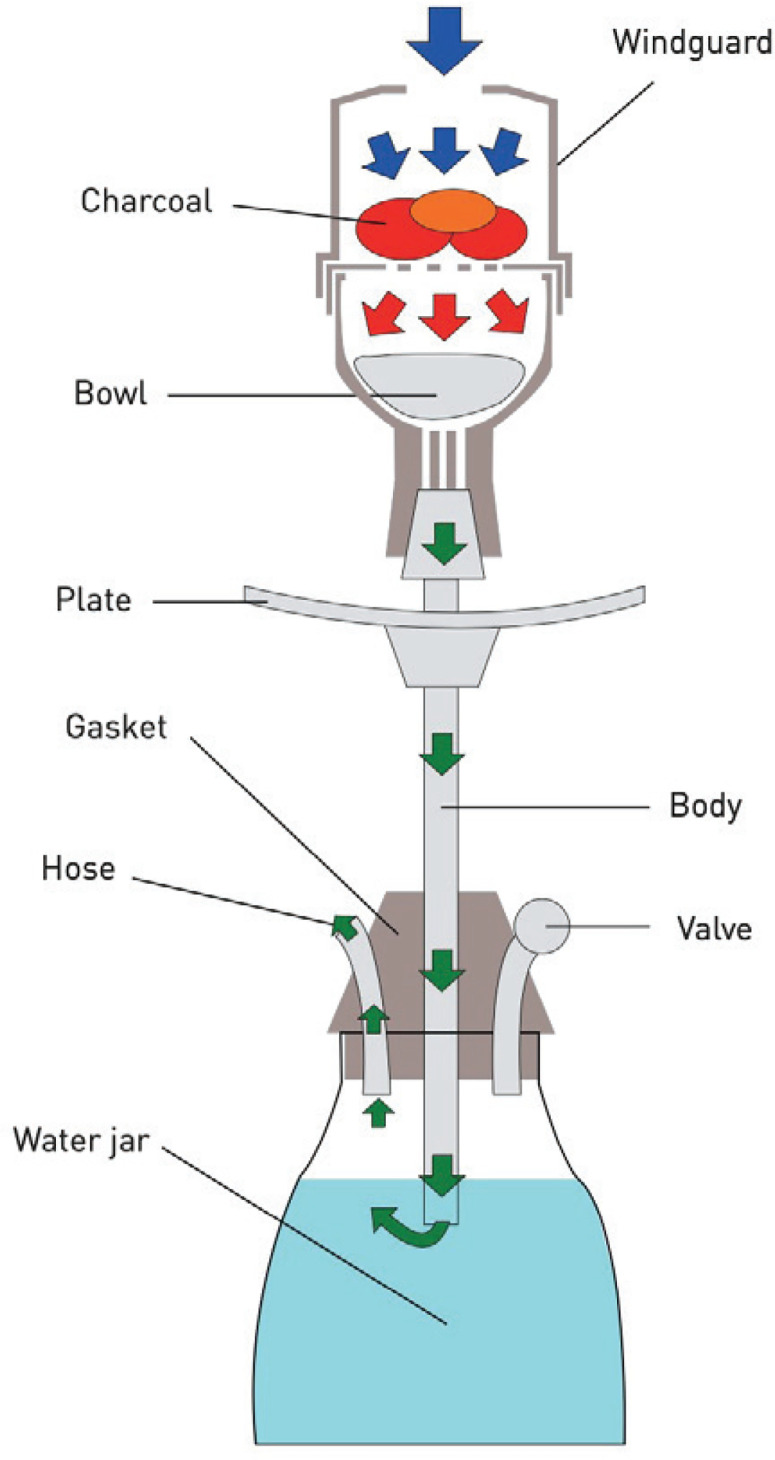
A description of a waterpipe instrument

WPS is gaining popularity worldwide. The waterpipe instrument is composed of several parts: 1) A head/bowl filled with flavored tobacco with charcoal added on top, with or without a perforated aluminum foil sheet that separates the tobacco from the charcoal; 2) A body composed of a metal pipe that connects the head/bowl to the base; and 3) A valved water-filled base with a hose that the smoker uses to inhale the smoke generated by the tobacco passes through the water in the base (Figure 2). The smoking sessions usually last for 0.5–1.5 hours. The volume of smoke generated during a single session was estimated to be 50–100 times that generated from a single cigarette^8^.

Despite the fact that WPS has been proven to induce oral cancer, lung cancer, cardiovascular disease, respiratory disease, and low birth weight, both smokers and non-smokers still believe that WPS is less harmful than cigarettes, which is most likely due to the relaxing, socializing, and pleasant environment associated with it^22,23^. In addition, effective policies addressing the harmful effects of WPS have not been as well established as policies against cigarette smoking^24^.

For decades, WPS has been dominated by male smokers, but due to the emergence of WPS cafés that provide perfect settings for social gatherings, especially in the Middle East, it has become popular among both genders^25^. Moreover, differing perceptions in different countries and societies with regard to gender roles seem to influence women’s WPS habits^26^.

Previous prevalence studies in Kuwait have shown that WPS is higher in women than in men, similar to the current study^5^. More than half of males and females have smoked waterpipes at least once in their lives in Kuwait, and most began using them in their teens^5^. Across several populations worldwide, relatively younger cohorts and adolescents have taken up hookah smoking^2,27^. In our study, half of the waterpipe smokers belonged to the third decade, and the percentage of smokers decreased with a further increase in age. This is in accordance with the findings of Mohammad et al.^7^ who showed that non-smokers were more likely to be older. Social acceptance, peer influence, perception of looking ‘cool’, and physiological effects commonly referred to as ‘buzz’ were the main reasons for the initiation of WPS at a young age^28^.

In this study, most Kuwaitis were shisha smokers. Previous studies have also shown that smoking (both shisha and cigarettes) is widely prevalent among Kuwaiti citizens^5,7^. However, the fact that this study was conducted in venues frequently visited by Kuwaiti citizens should be considered.

Most smokers in this study had a high level of education (high school or above). Despite a high level of education, this unhealthy behavior was adopted. Similar findings were reported in Saudi Arabia, where there was a high prevalence of shisha smoking among university students^29^. A previous study in Kuwait reported that waterpipe smokers had more positive beliefs regarding WPS and were less knowledgeable about the health effects of waterpipe smoking^7^. Hence, awareness programs should aim to correct inaccurate beliefs while reinforcing facts regarding WPS, irrespective of their prior education level.

Generally, NS exists without symptoms or is mildly irritating. Most often, patients report that they are either oblivious to the lesion or that they have noticed the lesion without any changes over several years. NS is reversible and has an excellent prognosis after the irritant is eliminated^13^. Despite the fact that tobacco smoking causes NS, it is generally not associated with dysplastic or neoplastic alterations^14^. It has the same malignant potential as a normal hard and soft palate^13^. However, in reverse smokers, the concentrated heat and chemicals raise the risk for malignant changes^30^. Thus, NS is a sign of heavy tobacco use. Patients with NS require a thorough oral examination as premalignant and malignant mucosal lesions can develop on other high-risk mucosal surfaces^14^.

In our study, approximately a quarter (26.1%) of participants had NS. However, it is interesting to note that only 37 (35.2%) had NS among shisha smokers, whereas 68 (64.8%) did not. In addition, all cases of NS detected were mild. Moreover, males were 2.28 times more likely to present with NS. Twentytwo (40%) participants with NS were male smokers, whereas only 15 (27.3%) with lesions were female smokers.

Previous studies from Iraq and Indonesia have shown that none of the waterpipe smokers developed NS^31,32^. In a study from Saudi Arabia on the effects of tobacco on oral health, 38.1% of participants were shisha users and showed oral lesions, including NS (28.9%)^33^.

Most smokers in this study preferred unflavored tobacco. In a study that analyzed commercial waterpipe tobacco, unflavored tobacco contained approximately 10 times more nicotine per gram than flavored tobacco. While the nicotine content is equivalent to 6.5 regular cigarettes in flavored tobacco, it is equivalent to 70 cigarettes for unflavored tobacco^34^. Al-Mutairi et al.^35^ showed that long-term waterpipe smokers (>10 years) absorb more nicotine than short-term waterpipe smokers (≤10 years).

All 55 participants with NS consumed hot beverages. All 37 smokers with lesions consumed hot beverages. This suggests that the high temperatures of hot drinks can be synergistic with the damage caused by tobacco compounds in waterpipe smoking. Earlier, dos Santos and Katz^11^ suggested a positive correlation between heat in maté tea drinks and smoking. None of the participants (smokers as well as non-smokers) who did not drink hot beverages had NS. However, as the total number of non-drinkers in the study was seven, a statistically significant association could not be derived. Further studies are required to confirm the additive effects of hot drinks and WPS.

In hot beverage drinkers with NS, the percentage of participants with lesions increased with an increase in the number of cups of hot beverages and the perceived heat rate of the beverage.

Measuring the heat and nicotine concentration generated by hookah is considered difficult for the following reasons: 1) Hookahs are heated by burning charcoal, and hookah smokers differ in their preference for the amount and size of charcoal used to heat tobacco products. Thus, the heat generated by hookah differs for everyone; 2) Different brands manufacture tobacco used in hookahs and have many flavors and different concentrations of nicotine, in addition to other additives, thus unifying these variables would be challenging for researchers; 3) Puff topography (number of puffs drawn, puff volume, duration of puffs, and interval between consecutive puffs) is a difficult variable to measure because the consumption time of a single serving differs among hookah smokers. Nonetheless, an average hookah smoker would consume a single serving in 60 min. Other smokers might take more than an hour per round or serving, depending on the puffing rate; and 4) Hookah design and construction are not standardized, further complicating measuring heat and nicotine generated by such devices.

Therefore, the participants were asked about their preferences for mild, moderate, or high heat generated by their hookahs, their smoking hours per day, and other details.

The number of hours a shisha was smoked was found to be significantly higher among shisha smokers with NS, as well as the number of tobacco heads smoked per day.

### Limitations

The major limitation of the current study is the method of non-random convenient sampling that can lead to sampling bias and observer bias. However, to reduce bias, the research was conducted in different places at different times over several months. The inclusion criteria were well defined, as mentioned in the Methods section. The non-causal study design limits the ability to draw conclusions about cause and effect. It should be borne in mind that the study is descriptive, and therefore correlation cannot imply causation. Comparisons to similar studies from other countries need to be done with caution as the recruitment of participants and methodologies differ. The current study is cross-sectional in design. Furthermore, the control of confounding factors was not exhaustive. Therefore, residual confounding in the study is inevitable.

## CONCLUSIONS

The current study reports that the prevalence of mild NS is related to WPS and also suggests evidence for the additive effect of hot beverages. Prevalence of NS was found to be associated with the increase in a number of tobacco heads smoked as well as an increase in the duration of smoking. To the best of our knowledge, this is the first study to explore the relationship between waterpipe smoking and NS. Given the cross-sectional nature of this study, a causal relationship between shisha smoking and NS could not be established. Further longitudinal studies on the effects of WPS will provide a better understanding of this phenomenon. Currently, dentists should not disregard the use of these products by their dental patients. Dental care professionals, especially in the Middle East, where WPS is widely prevalent, should record its use in health records, present oral and general health effects to their patients, and provide counseling regarding tobacco cessation.

## Supplementary Material



## Data Availability

The data supporting this research are available from the authors on reasonable request.
