# Supplementary material for: The association between nicotine stomatitis and waterpipe smoking
Source: Tob Induc Dis. 2024 Jun 27;22:10.18332/tid/189600. doi: 10.18332/tid/189600 (PMC11201228; doi:10.18332/tid/189600)
Supplement: Supplementary file 1 [file TID-22-118-s1.pdf]

Supplementary Material

Supplementary Table 1. Criteria followed in current study to evaluate Nicotine Stomatitis severity

Palatal Changes Detected	Description
None	No changes are detected in the palate.
Mild	Focally or diffusely mild gray/white discoloration of the palatal mucosa and slightly raised papules centered by punctate red dots. The base of the raised papules measures less than 1 mm.
Moderate	Focally or diffusely moderate gray/white discoloration and hyperkeratosis of the palatal mucosa with raised papules that can be joined or separated, centered by punctate red dots. The base of the raised papules measures more than 1 mm and less than 2 mm.
Severe	Diffusely severe white discoloration and hyperkeratosis of the palatal with raised papules that can be joined or separated, centered by punctate red dots. The base of the raised papules measures more than 2 mm.

Supplementary Table 2. Number and percentage of patients with lesions with respect to hot beverage intake

Amount of hot beverage	Patients with Lesions n(%)
Less than a cup<br>One cup<br>2 cups<br>Others	4 (7.3)<br>10 (18.2)<br>20 (36.4)<br>21 (38.2)
Total	55 (100)
Perceived heat rate of beverage	Patients with Lesions n(%)
Warm<br>Hot	15 (27.3)<br>40 (72.7)
Total	55 (100)

©2024 Dashti H. and Sundaram D.
